# Is *N*1-Methylnicotinamide a Good Organic Cation Transporter 2 (OCT2) Biomarker?

**DOI:** 10.3390/metabo15020080

**Published:** 2025-01-29

**Authors:** Anoud Sameer Ailabouni, Gautam Vijaywargi, Sandhya Subash, Dilip Kumar Singh, Zsuzsanna Gaborik, Bhagwat Prasad

**Affiliations:** 1 Department of Pharmaceutical Sciences, Washington State University, Spokane, WA 99202, USA; anoud.ailabouni@wsu.edu (A.S.A.); gautam.vijaywargi@wsu.edu (G.V.); sandhya.subash@wsu.edu (S.S.); dilip.singh@wsu.edu (D.K.S.); 2 Charles River Laboratories Hungary Kft, H-1117 Budapest, Hungary; zsuzsanna.gaborik@crl.com

**Keywords:** renal transport, hepatic transport, organic cation transport, pharmacokinetic, drug–drug interactions, endogenous biomarkers

## Abstract

**Background/Objectives:** The impact of potential precipitant drugs on plasma or urinary exposure of endogenous biomarkers is emerging as an alternative approach to evaluating drug–drug interaction (DDI) liability. *N*1-Methylnicotinamide (NMN) has been proposed as a potential biomarker for renal organic cation transporter 2 (OCT2). NMN is synthesized in the liver from nicotinamide by nicotinamide N-methyltransferase (NNMT) and is subsequently metabolized by aldehyde oxidase (AO). Multiple clinical studies have shown a reduction in NMN plasma concentration following the administration of OCT inhibitors such as cimetidine, trimethoprim, and pyrimethamine, which contrasts with their inhibition of NMN renal clearance by OCT2. We hypothesized that OCT1-mediated NMN release from hepatocytes is inhibited by the administration of OCT inhibitors. **Methods:** Re-analysis of the reported NMN pharmacokinetics with and without OCT inhibitor exposure was performed. We assessed the effect of cimetidine on NMN uptake in OCT1-HEK293 cells and evaluated the potential confounding effects of cimetidine on enzymes involved in NMN formation and metabolism. **Results:** A re-analysis of previous NMN pharmacokinetic DDI data suggests that NMN plasma systemic exposure decreased by 17–41% during the first 4 h following different OCT inhibitor administration except dolutegravir. Our findings indicate that NMN uptake was significantly higher (by 2.5-fold) in OCT1-HEK293 cells compared to mock cells, suggesting that NMN is a substrate of OCT1. Additionally, our results revealed that cimetidine does not inhibit NNMT and AO activity. **Conclusions:** Our findings emphasize the limitations of using NMN as an OCT2 biomarker and reveal potential mechanisms behind the reduction in NMN plasma levels associated with OCT inhibitors. Instead, our data suggest that NMN could be tested further as a potential biomarker for OCT1 activity.

## 1. Introduction

The effect of transporters on drug disposition, safety, and efficacy is now well-recognized [1]. The kidneys play a crtitical lrole in the elimination of drugs, environmental compounds, and endogenous metabolites into urine [2]. In addition to passive glomerular filtration, these compounds can be eliminated into urine through active drug transport systems (involving tubular secretion and reabsorption) in the proximal renal tubules [2]. Renal tubular secretion is primarily facilitated by cation transporters expressed in the proximal tubules. Organic cation transporter 2 (OCT2), located on the basolateral membrane of human kidneys, uptakes cation substrates from the blood into renal tubular cells. These substrates are then effluxed into urine through multidrug and toxin extrusion 1 (MATE1) and MATE2K transporters expressed in the renal apical membrane [3]. Inhibition of OCT2 and/or MATE1/2K can decrease overall renal clearance (CL_r_) and elevate plasma exposure to substrate compounds, leading to potential safety issues [4].

The use of endogenous biomarkers is a rapidly evolving field of research for monitoring transporter function and assessing the transporter-mediated drug–drug interaction (DDI) potential of a new molecular entity (NME) without the need to administer probe drugs. An ideal transporter biomarker must exhibit sensitivity, selectivity, and independence from confounding factors such as its formation or degradation or external factors such as diet [5]. According to the United States Food and Drug Administration (FDA) and the European Medicines Agency (EMA), the biomarker-informed decision process for assessing renal DDI risk involves analyzing endogenous biomarker concentrations in plasma and urine during single ascending dose or multiple ascending dose trials in Phase I [6]. A significant increase in biomarker plasma systemic exposure by more than 1.25-fold or a significant reduction in its CL_r_ to less than 0.8-fold indicates potential inhibition of renal transporters and necessitates projecting DDI study using probe drug (i.e., metformin in case of OCT2) [6,7]. While multiple OCT2 endogenous substrates are proposed as its biomarkers, their utility in predicting renal DDI remains questionable and requires further investigation. *N*1-Methylnicotinamide (NMN) is the most investigated renal OCT2 biomarker, which is currently classified as a Tier 2 biomarker for OCT2, indicating that it is not yet fully validated as an OCT2 biomarker [1].

**Figure 1:**
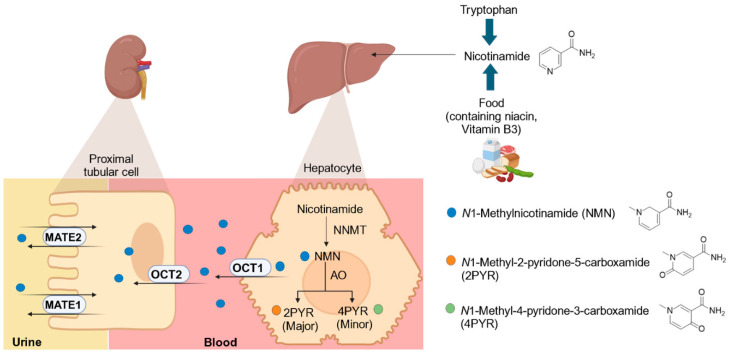
Schematic presentation of the formation, metabolism, and release of *N*1-methylnicotinamide (NMN) in the liver. NMN is synthesized in the liver from nicotinamide through the enzymatic action of nicotinamide N-methyltransferase (NNMT). NMN undergoes further metabolism catalyzed by aldehyde oxidase (AO), forming *N*1-methyl-2-pyridone-5-carboxamide (2PYR) and *N*1-methyl-4-pyridone-3-carboxamide (4PYR). NMN is then released into the systemic circulation through hepatic organic cation transporter 1 (OCT1). NMN is eliminated in the urine primarily through active tubulure secretion mediated by OCT2 and multidrug and toxin extrusion 1 (MATE1) and MATE2K transporters. The figure was created using BioRender.

NMN is primarily synthesized in the liver from nicotinamide through the enzymatic action of nicotinamide N-methyltransferase (NNMT) as part of nicotinamide metabolism [8]. NMN subsequently undergoes further metabolism catalyzed by aldehyde oxidase (AO), forming *N*1-methyl-2-pyridone-5-carboxamide (2PYR) as a major metabolite and *N*1-methyl-4-pyridone-3-carboxamide (4PYR) (Figure 1) [8]. The CL_r_ of NMN is two–six times higher than the glomerular filtration rate (GFR), suggesting the involvement of renal secretion [9]. NMN has been identified as an endogenous probe for renal cation transporters after demonstrating a decrease in CL_r_ of NMN and metformin in healthy volunteers treated with trimethoprim, an OCT2/MATE inhibitor [10]. Reliable potential renal transporter biomarkers are anticipated to demonstrate a reduction in CL_r_, accompanied by either an increase in systemic plasma exposure (e.g., creatinine) [11] or no change (e.g., *N*1-methyladenosine) [12] when coadministered with precipitants. Interestingly, analysis of clinical DDI study data from our laboratory [13] showed a decrease in the plasma concentration of NMN after cimetidine treatment in healthy adults. This finding aligns with several studies using different OCT precipitants, such as trimethoprim [10], cimetidine [11,14], and pyrimethamine [9]. While the role of NMN as an OCT2 biomarker has been extensively studied, its potential plasma reduction following OCT inhibitor administration remains underexplored.

In this study, we aim to evaluate the potential role of hepatic OCT1 in influencing NMN plasma exposure. We hypothesize that OCT1 regulates NMN release from hepatocytes into the systemic circulation, and its inhibition may lead to a decrease in NMN plasma concentrations, as observed in clinical studies [9,10,11,13,14]. To test this hypothesis, we performed an in vitro uptake assay to assess NMN transport by OCT1 and examined the effect of cimetidine on this transport activity. To exclude the possibility of metabolic interference, we also investigated the impact of cimetidine on the enzymatic activities of NNMT and AO. Our findings suggest that NMN is a substrate of OCT1, which can be tested as a potential biomarker.

## 2. Materials and Methods

### 2.1. Chemicals and Reagents

Metformin hydrochloride, cimetidine, metformin-d6, and *N*1-methylnicotinamide-d3 iodide were purchased from Toronto Research Chemicals (Toronto, ON, Canada). LC–MS grade of methanol, acetonitrile, water, formic acid, dimethylsulfoxide (DMSO), and buffer salts (potassium dihydrogen phosphate and dipotassium hydrogen phosphate) were procured from Fisher Scientific (Fair Lawn, NJ, USA). Bovine serum albumin (BSA), nicotinamide, carbazeran, Trizma^®^ base, and Trizma^®^ hydrochloride were purchased from Sigma-Aldrich (St. Louis, MO, USA). *N*1-Methylnicotinamide chloride, recombinant human nicotinamide-N-methyltransferase (rhNNMT), and *S*-(5′-Adenosyl)-*L*-methionine (AdoMet) sulfate tosylate were purchased from Cayman Chemical (Ann Arbor, MI, USA). Pooled human liver cytosol (HLC, mixed gender; n = 50) was purchased from Xenotech (Kansas, MO, USA). Human serum albumin (HSA) was purchased from Calbiochem (Billerica, MA, USA). Stably transfected human embryonic kidney 293 (HEK293)-mock-B-LV cells (transfected with empty vector) and HEK293 cells overexpressing organic cation transporter 1 (OCT1) were obtained from Charles River Laboratories, Solvo Biotechnology (Szeged, Hungary). Dulbecco’s modified Eagle’s medium (DMEM), fetal bovine serum (FBS), penicillin and streptomycin solution, and blasticidin were obtained from Invitrogen (Carlsbad, CA, USA). Gibco™ phosphate-buffered saline (PBS) 1X; pH 7.4 was purchased from Thermo Fisher Scientific (Waltham, MA, USA). BioCoat™ 24-well poly-D-lysine coated plates were purchased from Corning Inc. (Corning NY, USA). Hank’s balanced salt solution (HBSS) was acquired from Lonza Inc. (Allendale, NJ, USA). Dithiothreitol (DTT) was purchased from Thermo Scientific (Rockford, IL, USA)

### 2.2. A Literature Analysis of the Effect of OCT Inhibition on NMN Plasma Concentration

The plasma concentration and CL_r_ data on the effects of OCT inhibitors on NMN pharmacokinetics were curated from the literature studies, i.e., trimethoprim [10], cimetidine [11,13,14], pyrimethamine [9], and dolutegravir [14]. In particular, we examined the effects of (i) a single 400 mg oral dose of cimetidine, (ii) multiple 400 mg oral doses of cimetidine (5 times a day), (iii) a 200 mg oral dose of trimethoprim administered twice daily for 5 days, (iv) a single 50 mg oral dose of pyrimethamine, and (v) a 50 mg oral dose of dolutegravir twice daily on NMN pharmacokinetics in healthy adults.

In the clinical study performed in-house [13], we assessed the effect of a single 400 mg dose of cimetidine on NMN plasma and urine concentrations, and the full pharmacokinetic profile was determined using non-compartmental analysis in MATLAB^®^ (R2024b; Natick, MA, USA). For the other studies, we digitized clinical data using WebPlotDigitizer Version 4.8 (https://apps.automeris.io/wpd4/ (accessed on 23 November 2024)). Pharmacokinetic measures, including the area under the plasma concentration–time curve (AUC) at three-time intervals of 0–4, 0–12, and 0–24 h and maximum plasma concentration (C_max_), were obtained from the studies mentioned above or reanalyzed using non-compartmental analysis in MATLAB^®^ (R2024b; Natick, MA, USA). The reported CL_r_ of NMN in the above studies was considered as such.

### 2.3. N1-Methylnicotinamide Uptake by HEK293-OCT1-Transfected Cells

Stably-transfected mock and OCT1 overexpressing HEK293 cells were cultured in DMEM with 10% FBS, 100 U/mL penicillin, 100 µg/mL streptomycin, and 10 mg/mL blasticidin (selecting agent) and seeded at a density of 0.5 × 10^6^ cells/well on BioCoat™ 24-well poly-D-lysine coated plates. Cells were maintained at 37 °C and 5% CO_2_ for 16–20 h to ensure proper cell adhesion and monolayer formation. On the experiment day, all media was aspirated, and cells were washed twice with 300 µL of pre-warmed PBS buffer (pH 7.4). Following the second wash, an additional 300 µL of pre-warmed PBS buffer was added, and cells were incubated for 15 min at 37 °C and 5% CO_2_ to equilibrate. The buffer was then aspired, and the uptake assay was initiated by adding 200 µL of a reaction mixture containing 50 µM NMN in HBSS and incubating at 37 °C for 5 min. After the incubation period, uptake was terminated by aspirating the entire reaction mixture and rinsing the cells twice with 300 µL of ice-cold PBS buffer. After aspirating the second wash, cells were lysed by adding 600 µL of acetonitrile containing NMN-d3 (50 ng/mL) and metformin-d6 (50 ng/mL) as an internal standard. The plate was kept at 4 °C for 20 min to facilitate cell lysis. Subsequently, 550 µL of cell lysate sample was transferred into a microcentrifuge tube after appropriate pipette mixing. Samples were then centrifuged at 16,000× *g* at 4 °C for 10 min, and 520 µL of the supernatant was transferred into a different tube and evaporated at 45 °C until completely dried using Concentrator plus/Vacufuge plus (Eppendorf, Germany). Dried samples were reconstituted with 200 µL of 0.1% formic acid in acetonitrile: water (10:90) and vortex for 5 min. Samples were centrifuged at 16,000× *g*, 4 °C for 10 min, and then, 15 µL of the supernatant was transferred into a vial for quantification of NMN uptake into HEK293 cells overexpressing OCT1 versus mock HEK293 cells.

### 2.4. Quantification of OCT1 Uptake of NMN in the Presence and Absence of Cimetidine Using HEK293 Overexpressing OCT1

In vitro OCT1 uptake assay of NMN and metformin (positive control) was evaluated with and without cimetidine in two separate 24-well plates. A similar protocol was used as described above. Cimetidine solutions (200 µL) or reaction buffer (200 µL) containing 0.71% *v*/*v* DMSO (0, 1, 10, 100, and 200 µM of cimetidine) were added to cells, and the plates were incubated for 5 min at 37 °C before the addition of substrate reaction mixture. After a 5-min incubation time of cells with cimetidine, cimetidine solutions were removed, and the reaction mixture containing 50 µM NMN or 100 µM metformin with cimetidine (0, 1, 10, 100, and 200 µM) was added. The plates were then incubated at 37 °C for an additional 5 min.

### 2.5. Quantification of Carbazeran Metabolism by Aldehyde Oxidase in the Presence and Absence of Cimetidine Using Human Liver Cytosol (HLC)

The aldehyde oxidase (AO) catalyzed conversion of carbazeran to 4-oxo-carbazeran (positive control) in the presence and absence of cimetidine was determined in pooled HLC containing 0.1 M potassium phosphate buffer (pH 7.4). All incubations were carried out in triplicate in a water bath shaker at 37 °C, with final reaction volumes of 100 µL each. The content of AO in all incubations was maintained at 2 pmol, and the total protein content was adjusted to 174 µg using HSA [15]. Cimetidine (5 µL) or reaction buffer (5 µL) was then added to achieve final cimetidine concentrations of 0, 1, 10, and 100 µM, and the reaction mixture was incubated for 5 min at 37 °C. The final concentration of DMSO in all incubations was 0.35% *v*/*v*, which has been reported not to affect AO activity [16]. Reactions were initiated by adding 5 µL of carbazeran in each incubation to achieve a final concentration of 25 µM. The reactions were terminated after 5 min by adding 200 µL acetonitrile containing 500 ng/mL NMN-d3 (internal standard). Samples were centrifuged at 10,000× *g* for 10 min at 4 °C, and 50 µL of supernatant was mixed with 50 µL of 0.1% formic acid in acetonitrile:water mix (5:95, *v*/*v*). These solutions were then transferred to LC vials for analysis of the oxidative metabolite of carbazeran (4-oxo-carbazeran).

### 2.6. Quantification of NMN Formation by NNMT in the Presence and Absence of Cimetidine Using Recombinant Human NNMT

Enzyme activity assays were performed with recombinant human NNMT (rhNNMT) (15.4 μg/mL) in 50 mM Tris buffer (pH 8.6, prepared from Trizma^®^ base and Trizma^®^ hydrochloride) containing 1 mM DTT in the presence or absence of cimetidine. All incubations were carried out in triplicate in a water bath shaker at 37 °C with incubation volumes of 100 µL each. Briefly, rhNNMT (5 μL of 0.308 mg/mL) and nicotinamide (5 μL of 20.5 mM) were added to 80 μL of 50 mM Tris buffer (pH 8.6) containing 1 mM DTT, resulting in final concentrations of 15.4 µg/mL rhNNMT and 1 mM nicotinamide. Cimetidine (5 μL) or reaction buffer (5 μL) was then added to achieve final cimetidine concentrations of 0, 1, 10, and 100 µM and incubated for 5 min at 37 °C. The final concentration of DMSO in all incubations was 0.35% *v*/*v*, which has been reported not to affect NNMT activity [17]. The enzyme reactions were initiated by adding 5 µL AdoMet (methyl donor cofactor) to attain a final concentration of 40 µM in the reaction mixture and incubated for 30 min at 37 °C. The reactions were terminated by adding 200 µL of acetonitrile containing 500 ng/mL NMN-d3 (internal standard). Samples were centrifuged at 10,000× *g* for 10 min at 4 °C, and 50 µL of supernatant was mixed with 50 µL of 0.1% formic acid in acetonitrile:water mix (5:95, *v*/*v*). These solutions were then transferred to LC vials for the analysis of metabolite formation of nicotinamide metabolite (NMN).

### 2.7. Targeted LC–MS/MS Analysis of Metabolites

All in vitro samples were analyzed using a validated LC–MS/MS method consisting of an M-Class microflow Acquity UPLC^®^ coupled with Xevo-TQ-XS MS (Waters, Milford, MA, USA). Chromatographic separation of the analytes was achieved using an Acquity UPLC^®^ HSS T3 (1.8 µm, 1 × 100 mm) column (Waters, Milford, MA, USA). The mobile phase consisted of 0.1% formic acid in water (A) and 0.1% formic acid in acetonitrile (B), operated using different gradient programs according to the experiment and substrate of interest (Table S1). The mobile phase flow rate was set at 50 µL/min, and the injection volume was 1 µL. MS ionization was performed using an electrospray ionization source (ESI), and samples were analyzed using positive ionization and multiple reaction monitoring (MRM) modes. The MRM transitions were as follows: metformin (*m*/*z* 130.1→60.1, 70.1, and 88.1), metformin-d6 (*m*/*z*: 136.1→77.1 and 94.1), NMN (*m*/*z* 137.1→94.1), NMN-d3 (*m*/*z* 140.1→97.1), and 4-oxo-CBZ (*m*/*z* 377.3→288.1). Detailed LC–MS/MS parameters, including MRM transitions and collision energies, are provided in Table S2. LC–MS/MS peak integration and quantification were performed using Skyline software 23.1.0.268. (University of Washington, Seattle, WA, USA).

### 2.8. Structural Confirmation of Metabolites Using High Resolution-Mass Spectrometry

Carbazeran and nicotinamide incubated with AO and NNMT, respectively, were analyzed to confirm the 4-oxo-carbazeran and NMN formations using a nano-flow-based liquid chromatography-tandem high-resolution mass spectrometry system (nanoLC-HRMS/MS, Thermo Scientific™ Q Exactive™ HF). Chromatographic separation of metabolites was achieved using a Thermo Scientific PepMap™ RSLC C18 column (75 µm × 25 cm, 2 µm). The mobile phase consisted of 0.1% formic acid in water (A) and 0.1% formic acid in 80% acetonitrile (B), and the following gradients were applied: 0–1 min (0% B); 1–11 min (10% to 30% B); 11–16 min (30% to 50% B); 16–25 min (50% to 100% B); and 25–40 min (100% B) with mobile phase flow rate of 300 nL/min. The injection volume and column temperature were set to 1 µL and 40 °C, respectively. MS ionization was achieved using an EASY-spray ionization source with positive polarity. The samples were analyzed in Full MS and parallel reaction monitoring (PRM) modes to detect and confirm the metabolites, respectively. The MS was operated in full scan mode with an *m*/*z* scan range of 100–600, a resolution of 120,000, a capillary temperature of 300 °C, and a maximum injection time of 150 ms. PRM data were acquired at a resolution of 30,000 with an isolation window of 2.0 *m*/*z*, using stepped collision energies of 10, 20, and 30 eV to generate mass fragmentation of detected metabolites.

### 2.9. Statistical Analysis

The effect of cimetidine on the uptake and metabolism of NMN was analyzed using an unpaired two-tailed Student’s *t*-test using GraphPad Prism 8.4.3 (San Diego, CA, USA), and a *p*-value of <0.05 was considered statistically significant. The acceptance criteria for cimetidine as an inhibitor was a statistically significant reduction of more than 20% in samples containing cimetidine compared to control samples without cimetidine.

## 3. Results

### 3.1. Effect of OCT Inhibition on NMN Pharmacokinetics

**Figure 2:**
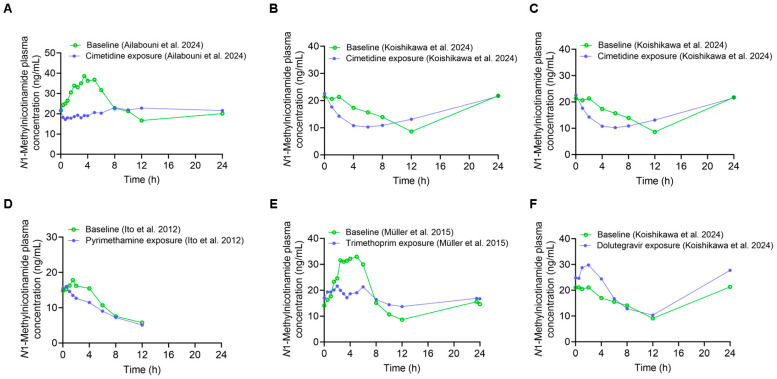
Effect of cimetidine, pyrimethamine, trimethoprim, and dolutegravir (OCT inhibitors) on *N*1-methyl nicotinamide (NMN) plasma concentrations in healthy adults. Plasma concentration–time profiles of NMN with and without cimetidine exposure (Data from Ailabouni et al., 2024 [13] (**A**), Data from Müller et al., 2023 [11] (**B**), and Data from Koishikawa et al., 2024 [14] (**C**)); pyrimethamine exposure (Data from Ito et al., 2012 [9] (**D**)); trimethoprim exposure (Data from Müller et al., 2015 [10] (**E**)); and dolutegravir exposure (Data from Koishikawa et al., 2024 [14] (**F**)). NMN plasma concentrations in absence of OCT inhibitor (baseline) are indicated with green open circles, while NMN plasma concentrations in presence of OCT inhibitors are indicated with blue solid circles.

NMN plasma concentrations were significantly lower in the cimetidine exposure arm compared to the baseline arm in our study [13] (Figure 2A). Similar results were observed for cimetidine (Figure 2B,C), pyrimethamine (Figure 2D), and trimethoprim (Figure 2E) from other reported clinical studies [9,10,11,14]. Relative to baseline, the pharmacokinetic measurements of NMN showed a trend of reduction in plasma exposure and C_max_ following OCT inhibitor treatment in healthy adults (Table 1). In particular, the AUC_0–4 h_ was reduced by 17–41% with the treatment with cimetidine, pyrimethamine, and trimethoprim without affecting plasma levels after 12 h. In contrast, dolutegravir is the only OCT inhibitor that demonstrated a 20–37% increase in NMN plasma concentrations at all time intervals [14] (Figure 2F, Table 1). Nevertheless, consistent with OCT2 inhibition in the kidneys, CL_r_ of NMN was reduced after OCT inhibitor exposure, except when cimetidine was given as a single 400 mg oral dose.

### 3.2. NMN Is a Substrate of OCT1; Cimetidine Inhibits NMN and Metformin Uptake Mediated by OCT1

**Figure 3:**
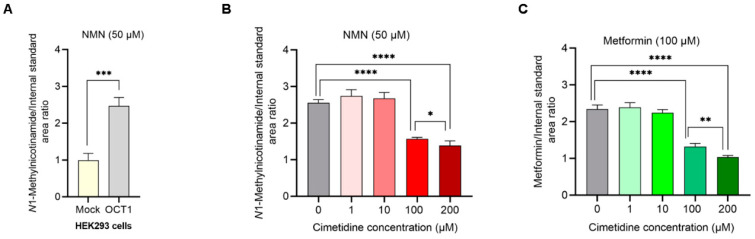
Inhibition of OCT1-mediated *N*1-ethylnicotinamide (NMN) transport by cimetidine in HEK293-OCT1 cell lines. Uptake of 50 µM NMN by HEK293 cells overexpressing OCT1 and mock HEK293 cells after 5 min incubation (**A**). Uptake of 50 µM NMN by HEK293 cells overexpressing OCT1 alone and with three different concentrations of cimetidine (1,10, 100, and 200 µM) (**B**). Uptake of 100 µM metformin (positive control) by HEK293 cells overexpressing OCT1 alone and with three different concentrations of cimetidine (1,10, 100, and 200 µM) (**C**). Each point represents mean ± SE (n = 4). Two-tailed unpaired *t*-test; *p*-value < 0.05 (*), <0.01 (**), <0.001 (***), and <0.0001 (****).

The uptake of NMN was significantly higher by 2.5-fold in OCT1 overexpressing HEK293 cell lines than in mock cells (Figure 3A). Cimetidine at 1 and 10 µM did not result in OCT1 inhibition, where metformin and NMN uptake in HEK293 cells overexpressing OCT1 was comparable with control cells without cimetidine. However, 100 µM of cimetidine showed a statistically significant reduction in NMN and metformin uptake by 39% and 43%, respectively (Figure 3B,C). Higher cimetidine concentration (200 µM) resulted in more reduction in NMN and metformin uptake by 46% and 56%, respectively, relative to their uptake by cells not exposed to cimetidine.

### 3.3. Cimetidine Does Not Affect AO-Mediated Metabolism of CBZ and NMN

**Figure 4:**
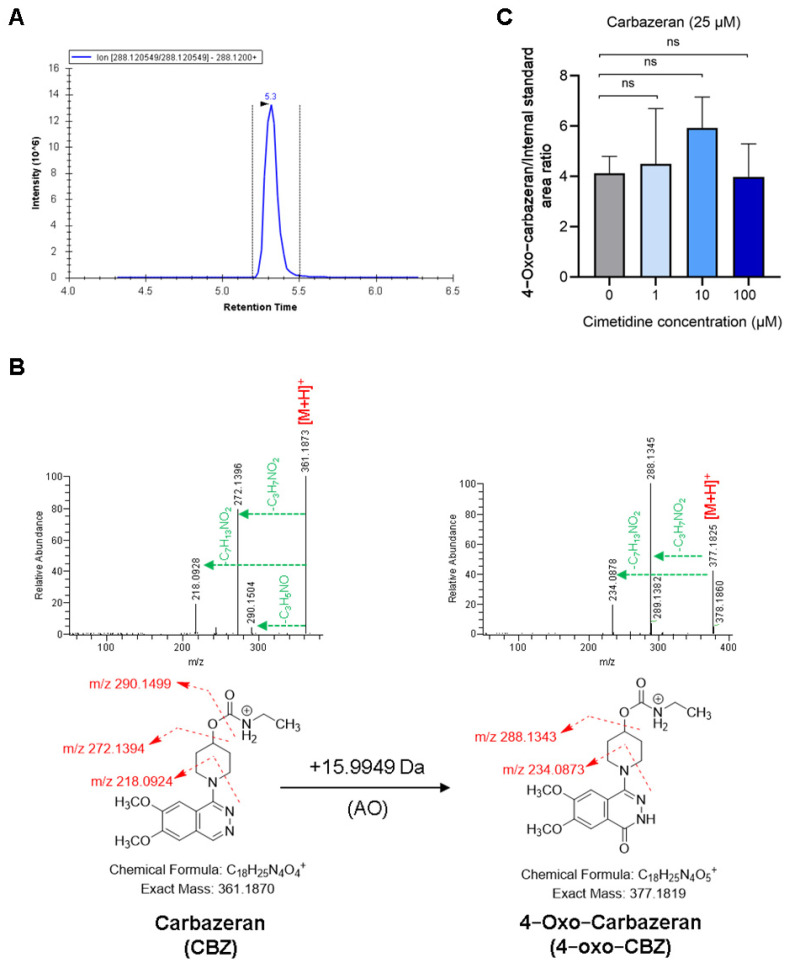
Effect of cimetidine on aldehyde oxidase (AO) activity. The 4-Oxo-carbazeran MRM signal intensity and retention time (**A**). Mass fragmentation pattern of carbazeran (CBZ) and its metabolite oxo-carbazeran (4-oxo-CBZ) in ESI positive mode. The exact masses of fragment ions (in red text) are depicted on the structure, while neutral losses are shown in the HR–MS/MS line spectra (green dotted lines) (**B**). Carbazeran metabolism to 4-oxo-carbazeran by AO in human liver cytosol alone and with three different concentrations of cimetidine (1, 10, and 100 µM) (**C**). In (**C**), each point represents mean ± SE (n = 3). Two-tailed unpaired *t*-test; *p*-value > 0.05 (ns, not significant).

The oxidation reaction of CBZ was confirmed by the formation of 4-oxo-CBZ, showing its MRM peak (Figure 4A). The HR–MS analysis revealed an accurate mass of 377.1825 Da, measuring 15.995 Da higher than that of CBZ (*m*/*z* 361.1873), indicating oxidative metabolism of CBZ to form 4-oxo-CBZ. In addition, the 4-oxo-CBZ displayed two major fragments of *m*/*z* 288.1345 and *m*/*z* 234.0878, each 16 Da higher than the corresponding CBZ fragments at *m*/*z* 272.1396 and *m*/*z* 218.0928 (Figure 4B), respectively. This comparative MS fragmentation pathway between CBZ and its metabolite supports oxidative metabolism occurring at the phthalazine ring of CBZ. In addition, the elemental composition calculator predicts the same ring plus double bond (RDB) value of 8.5 for protonated CBZ, and 4-oxo-CBZ further supports the same. Cimetidine at three different concentrations (1, 10, and 100 µM) did not inhibit the AO-catalyzed conversion of carbazeran (positive control) to 4-oxo-carbazeran (Figure 4C).

### 3.4. Cimetidine Does Not Affect NNMT Activity-Mediated Formation of NMN

**Figure 5:**
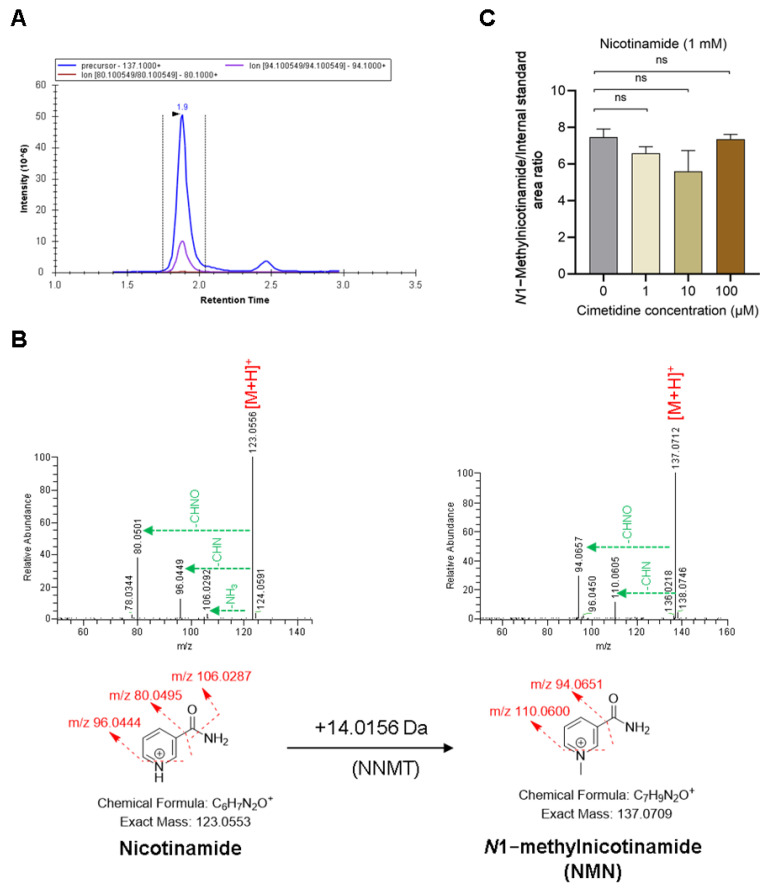
Effect of cimetidine on nicotinamide N-methyltransferase (NMNT) activity for *N*1-methylnicotinamide (NMN) formation. NMN MRM signal intensity and retention time (**A**). Mass fragmentation pattern of nicotinamide and its metabolite NMN in ESI positive mode. The exact masses of fragment ions (in red text) are depicted on the structure, while neutral losses are shown in the HR–MS/MS line spectra (green dotted lines) (**B**). Nicotinamide metabolism to NMN by using recombinant human NNMT alone and with three different concentrations of cimetidine (1, 10, and 100 µM) (**C**). In (**C**), each point represents mean ± SE (n = 3). Two-tailed unpaired *t*-test; *p*-value > 0.05 (ns, not significant).

The reaction of nicotinamide with rhNNMT was confirmed by the formation of NMN, showing its MRM peaks (Figure 5A). The HR–MS analysis showed an accurate mass of *m*/*z* 137.0712 (Figure 5B), corresponding to NMN, which was 14 Da higher than nicotinamide (*m*/*z* 123.0553), confirming methylation reaction. Furthermore, two characteristic NMN fragments *m*/*z* 110.0605 (loss of HCN) and *m*/*z* 94.0657 (loss of CHNO) were observed, each 14 Da higher than the corresponding nicotinamide fragments of *m*/*z* 96.0449 and *m*/*z* 80.0501 (Figure 5B), respectively, with the same neutral losses confirmed the methylation at the nitrogen atom on the pyridine ring of nicotinamide. The three concentrations of cimetidine (1, 10, and 100 µM) did not change NNMT activity where NMN formation of nicotinamide was comparable with and without cimetidine, as well as within the different concentrations of cimetidine (Figure 5C).

## 4. Discussion

Regulatory agencies like FDA, EMA, and the International Council for Harmonisation (ICH) [18] have issued guidelines for investigating the transporter inhibition potential of NMEs using transporter probe drugs in clinical studies. Although renal DDIs are typically mild, with an expected increase in plasma exposure by a maximum of four-fold [19], in vitro and in vivo studies to assess renal OCT2 interactions remain a prerequisite by regulatory agencies, with metformin recommended as a probe drug. The regulatory agencies recommend calculating the unbound maximum plasma concentration-to-half maximal inhibitory concentration ratio (C_max,u_/IC_50_) for OCT2 and MATEs transporters, as it quantitatively reflects in vivo inhibition potency. Conducting clinical DDI studies is warranted based on positive in vitro transporter inhibition results despite the possibility of false-positive data, reaching an approximate rate of 30% due to high inter-laboratory and inter-system variability in reported IC_50_ values [5,20]. Additionally, clinical DDI studies increase drug development costs and raise safety concerns due to the risk of elevated plasma levels of the administered exogenous probe drug resulting from severe DDI with NMEs [21]. Consequently, many academic and industry efforts have been directed at addressing the challenges of in vitro and clinical DDI studies. These efforts include using endogenous compounds as transporter biomarkers to assess in vivo transporter function and implementing emerging translational tools such as physiologically based pharmacokinetic (PBPK) models to predict transporter-mediated DDI potential [1].

NMN is reported as an OCT2 substrate based on various in vitro uptake assays conducted in HEK293 cells that stably expressed human OCT2 versus wild-type HEK293 cells [6,9,12,22]. Further, NMN has been postulated as a renal OCT2 biomarker following multiple studies that observed a decrease in NMN CL_r_ after administration of different perpetrators such as trimethoprim [10] and cimetidine [11]. Conversely, another study revealed no alteration in NMN plasma exposure and a significant decrease in NMN CL_r_ for pyrimethamine-treated healthy subjects compared to a control group, suggesting NMN as a potential MATE endogenous probe [9]. Other challenges associated with the use of NMN as an OCT2 endogenous probe include circadian rhythm variability in plasma exposure [23] and the involvement of NMN renal tubular reabsorption [24]. As a result, NMN is still not yet a fully validated OCT2 biomarker, and clinical data collection from Phase 1 studies is recommended despite NMN demonstrating high sensitivity and selectivity to OCT2 based on in vitro transport assays [1].

In our study, we wanted to investigate the reliability of using NMN as an OCT2 biomarker after observing a reduction in NMN plasma concentration in healthy adults after they were administered different OCT inhibitors, such as cimetidine. The clinical study conducted in our lab showed a 41% reduction in NMN plasma exposure during the first 4 h following the administration of 400 mg of cimetidine to healthy adults [13]. In alignment with our data, relative to the baseline, Koishikawa et al. [14] and Müller et al. [11] observed a reduction of 36% and 24% in NMN systemic exposure, respectively, during the time interval (0–4 h) following cimetidine treatment. Further, trimethoprim [10] and pyrimethamine [9] administration demonstrated a 23% and 17% reduction in NMN systemic exposure, respectively, within the first 4 h of precipitant treatment.

Given that the liver is the primary site of NMN synthesis due to the predominant expression of NNMT in hepatocytes [25] and that NMN is a cationic species requiring cation transporters for cellular uptake and efflux, we sought to investigate whether NMN was released into the bloodstream via OCT1, the major bidirectional cation transporter in the liver [26]. Our findings identified NMN as a substrate of OCT1, suggesting that following its synthesis in the liver, NMN was transported into systemic circulation mainly through OCT1. Consistent with our findings, a previous study reported NMN as a hepatic OCT1 substrate, showing an 11-fold increase in NMN uptake in OCT1-transfected HEK293 cells compared to mock HEK293 cells [22].

Further, our data demonstrated that cimetidine inhibited OCT1-mediated NMN uptake in a dose-dependent manner by 39% and 46% at 100 and 200 µM, respectively. Consistent with our data, cimetidine, trimethoprim, and pyrimethamine were reported to inhibit OCT1 with IC_50_ values of 275, 27.2, and 4.46 µM, respectively [27]. Since OCT1 is bidirectional [28], cimetidine administration may impair the release of NMN from the liver into systemic circulation, leading to a decrease in plasma NMN levels. Our findings support NMN as a potential OCT1 biomarker with higher sensitivity in detecting hepatic interaction (reduction in AUC) compared to renal OCT2 interaction (reduction in CL_r_). In contrast, dolutegravir exhibited the opposite effect to other OCT inhibitors, with NMN plasma concentrations showing an increasing trend across all time intervals [14]. The maximum increase (37%) was observed during the 0–4 h period following dolutegravir administration. This contradictory effect of dolutegravir might be attributed to its specific inhibitory effect toward OCT2 compared to the minimum inhibition of OCT1. Previous studies demonstrated that dolutegravir was not a clinically relevant inhibitor of OCT1 [29,30]. However, we would like to acknowledge that the potential inhibition of nicotinamide uptake into the liver by cimetidine could be an alternate mechanism contributing to the decrease in NMN levels observed with OCT inhibitors. However, the transporter responsible for nicotinamide uptake into hepatocytes has not been fully elucidated. Mathialagan et al. investigated the transport characteristics of nicotinamide in HEK293 cells transfected with various hepatic transporters, including OCT1, OCT3, NTCP, OATP1B1, OATP1B3, OAT1, and OAT2 [22]. The authors demonstrated that nicotinamide did not exhibit affinity for these transporters, suggesting that it was transported via an alternative mechanism [22]. Furthermore, another study identified nicotinamide as a substrate of Slc12a8, a transporter expressed in the small intestine, which contributes to its absorption [31]. While Slc12a8 is highly expressed in the small intestine, its expression in the liver is moderate [31]. Therefore, further studies are needed to understand the inhibition of nicotinamide uptake in human hepatocytes as an alternative mechanism to explain the observed decrease in NMN plasma concentrations with cimetidine and other inhibitors.

One study implied hypothetical NNMT inhibition in the NMN PBPK model by utilizing optimized values for the inhibitory constant of NMN synthesis (Ki,syn). The model successfully predicts NMN plasma concentration–time profiles with and without inhibitors [32]. We aimed to assess whether cimetidine inhibited the enzymatic activity of NNMT involved in NMN formation. Our data suggested that cimetidine, up to 100 µM, did not affect NNMT activity, consistent with findings by Koishikawa et al., where neither cimetidine (20 µM) nor dolutegravir (30 µM) reduced NMN formation, indicating no impact on NNMT activity [14]. Similarly, our data showed that cimetidine did not alter AO activity. Carbazeran is a probe substrate of AO [33], and cimetidine treatment (up to 100 µM) did not result in any change in 4-oxo-carbazeran formation; hence, cimetidine is not anticipated to affect NMN metabolism into 2PYR and 4PYR. In contrast with our data, cimetidine was reported to inhibit zaleplon metabolism to 5-oxo-zaleplon (known to be formed by aldehyde oxidase) in HLC with IC_50_ equal to 231 ± 23 µM [34]. However, it should be noted that cimetidine is considered a weak in vitro inhibitor of human AO [34,35]. Thus, our results suggest that cimetidine neither affects the formation nor the metabolism of NMN, potentially ruling out these mechanisms as causes for the observed decrease in NMN plasma exposure.

The efflux of endogenous NMN from hepatocytes in primary cell culture remains to be tested. However, multiple considerations should be taken into account for this experiment. For example, NMN is synthesized endogenously in hepatocytes, making it challenging to regulate its concentration across samples and accurately measure its retention or release. Further, OCT1 requires a negative membrane potential for its activity, which is difficult to replicate in an in vitro system.

## 5. Conclusions

Our study highlights the limitations of NMN as a sensitive and reliable biomarker for OCT2. Instead, we identified NMN as a substrate for OCT1, which can be evaluated for its potential utility as an OCT1 biomarker, particularly during early time points (AUC_0–4 h_). In vitro experiments show that cimetidine does not affect NMN formation or metabolism but inhibits OCT1-mediated NMN release into the systemic circulation. Additionally, several clinical studies provide further evidence that inhibiting hepatic OCT1 by administration of OCT inhibitors can reduce NMN plasma concentrations, which is consistent with our previous study.

## Figures and Tables

**Table 1:** Pharmacokinetics of *N*1-methylnicotinamide during baseline and exposure to different OCT inhibitors in healthy adults.

Pharmacokinetic Measures	Precipitant Dosing Regimen	AUC_0–24 h_ (µg·h/L)	AUC_0–12 h_ (µg·h/L)	AUC_0–4 h_ (µg·h/L)	C_max_ (µg/L)	CL_r_ (L/h)	Reference
Baseline	400 mg single oral dose	579.2	348.7	127.5	50.5	12.6	[13]
Cimetidine exposure		530.6	253.6	74.9	31.3	14.0	
Ratio (cimetidine exposure/baseline)		0.92	0.73	0.59	0.62	1.11	
Baseline	400 mg single oral dose	*370.9*	*188.6*	*80.7*	N/P	16.7	[14]
Cimetidine exposure		*359.6*	*151.4*	*61.1*	N/P	15.5	
Ratio (cimetidine exposure/baseline)		*0.97*	*0.80*	*0.76*		0.93	
Baseline	400 mg oral dose five times on day 1	*363.4*	220.8	*105.7*	31.5	17.4	[11]
Cimetidine exposure		*313.3*	155.0	*68.0*	28.8	12.6	
Ratio (cimetidine exposure/baseline)		*0.86*	0.70	*0.64*	0.91	0.72	
Baseline	200 mg oral dose twice daily for 5 days	396.9	*253.8*	*99.6*	40.1	15.5	[10]
Trimethoprim exposure		397.1	*212.9*	*77.0*	24.7	11.3	
Ratio (trimethoprim exposure/baseline)		*1.00*	0.84	*0.77*	0.62	0.73	
Baseline	50 mg single oral dose	N/A	136	*64.1*	N/P	24.18	[9]
Pyrimethamine exposure		N/A	114	*53.3*	N/P	7.14	
Ratio (pyrimethamine exposure/baseline)		N/A	0.84	*0.83*	N/P	0.30	
Baseline	50 mg oral dose twice daily on day 1	*371.0*	*188.3*	*79.9*	N/P	16.7	[14]
Dolutegravir exposure		*455.0*	*226.4*	*109.3*	N/P	14.0	
Ratio (dolutegravir exposure/baseline)		*1.23*	*1.20*	*1.37*	N/P	0.84	

AUC, area under the plasma concentration–time curve; C_max_, maximum plasma concentration; CL_r_, renal clearance; N/P, data not provided; N/A, not applicable because clinical study was conducted for 12 h duration. Italic font indicates pharmacokinetic data that were reanalyzed using MATLAB^®^ based on digitizing plasma concentration–time profiles.

## Data Availability

The authors declare that all the data supporting the findings of this study are contained within the paper.

## Supplementary Materials

The following supporting information can be downloaded at https://www.mdpi.com/article/10.3390/metabo15020080/s1, Table S1. Liquid chromatography (LC) method details for sample analysis; Table S2. Multiple reaction monitoring (MRM) method details for analysis of metformin, *N*1-methylnicotinamide (NMN), 4-oxo-carbazeran (4-oxo-CBZ), and internal standards.
