# Supplementary material for: Is N1-Methylnicotinamide a Good Organic Cation Transporter 2 (OCT2) Biomarker?
Source: Metabolites. 2025 Jan 29;15(2):80. doi: 10.3390/metabo15020080 (PMC11857448; doi:10.3390/metabo15020080)
Supplement: Supplementary file 1 [file metabolites-15-00080-s001.zip › metabolites-3366303-supplementary.pdf]

Title: Is *N*1-methylnicotinamide a good organic cation transporter 2 (OCT2) biomarker?

Authors: Anoud Sameer Ailabouni[1], Gautam Vijaywargi[1], Sandhya Subash[1], Dilip Kumar Singh[1], Zsuzsanna Gaborik[2], and Bhagwat Prasad[1,*]

Affiliation:

1. Department of Pharmaceutical Sciences, Washington State University, Spokane, WA
2. Charles River Laboratories, Solvo Biotechnology, Hungary

***Corresponding author:** Bhagwat Prasad, Ph.D., Department of Pharmaceutical Sciences, Washington State University, Spokane, WA 99202, USA. Phone: +1-509-358-7739. Fax: +1 509-368-6561. Email: bhagwat.prasad@wsu.edu

Table S1. Liquid chromatography (LC) method details for sample analysis

**LC Method and Parameters**			
	**Parameters**		
**LC**	M-class Waters UPLC		
**MS**	Waters Xevo TQ-XS		
**Column**	Acquity UPLC® HSS T3 (1.8 µm, 1x100 mm)		
**Mobile phase A**	0.1% Formic acid in water		
**Mobile phase B**	0.1% Formic acid in acetonitrile		
**Flow rate (µL/min)**	50		
**Column temperature (°C)**	40		
**Injection volume (µL)**	1		
**LC gradient program (I)**			
**Time (min)**	**%A**	**%B**	
**0.00**	95	5	
**1.00**	95	5	
**2.50**	45	55	**Analytes of interest:**
**4.20**	32	68	• Metformin
**5.20**	10	90	• *N*1-Methylnicotinamide
**8.00**	10	90	
**8.50**	95	5	
**11.50**	95	5	
**LC gradient program (II)**			
**Time (min)**	**%A**	**%B**	
**0.00**	95	5	
**1.00**	95	5	
**2.50**	45	55	**Analytes of interest:**
**4.20**	32	68	• 4-Oxo-carbazeran
**5.20**	10	90	
**8.00**	10	90	
**8.50**	95	5	
**11.50**	95	5	

Table S2. Multiple reaction monitoring (MRM) method details for analysis of metformin, *N*1-methylnicotinamide (NMN), 4-oxo-carbazeran (4-oxo-CBZ), and internal standards.

**MS method (Electrospray ionization (ESI), Positive mode)**					
**Compounds**	**Retention time (min)**	**Precursor *m/z***	**Product *m/z***	**Cone voltage (CV)**	**Collision energy (CE)**
Metformin	1.9	130.1	60.1	25	20
		130.1	70.1	22	17
		130.1	88.1	20	15
Metformin-d6	1.9	136.1	77.1	20	20
		136.1	94.1	20	15
*N*1-methylnicotinamide (NMN)	1.9	137.1	94.1	25	15
*N*1-methylnicotinamide-d3 (NMN-d3)	1.9	140.1	97.1	25	15
4-Oxo-carbazeran (4-oxo-CBZ)	5.3	377.3	288.1		
